# Selected Applications of the Theory of Connections: A Technique for Analytical Constraint Embedding

**DOI:** 10.3390/math7060537

**Published:** 2019-06-12

**Authors:** Hunter Johnston, Carl Leake, Yalchin Efendiev, Daniele Mortari

**Affiliations:** 1 Department of Aerospace Engineering, Texas A&M University, College Station, TX 77843, USA; 2 Department of Mathematics, Texas A&M University, College Station, TX 77843, USA

**Keywords:** linear constraint optimization, calculus of variation, over-constrained differential equations, inequality constraints, triangular domains, Theory of Connections

## Abstract

In this paper, we consider several new applications of the recently introduced mathematical framework of the Theory of Connections (ToC). This framework transforms constrained problems into unconstrained problems by introducing constraint-free variables. Using this transformation, various ordinary differential equations (ODEs), partial differential equations (PDEs) and variational problems can be formulated where the constraints are always satisfied. The resulting equations can then be easily solved by introducing a global basis function set (e.g., Chebyshev, Legendre, etc.) and minimizing a residual at pre-defined collocation points. In this paper, we highlight the utility of ToC by introducing various problems that can be solved using this framework including: (1) analytical linear constraint optimization; (2) the brachistochrone problem; (3) over-constrained differential equations; (4) inequality constraints; and (5) triangular domains.

## 1. Introduction

The Theory of Connections (ToC), introduced by D. Mortari [1] (see also [2–4]), is a framework that transforms constrained problems into unconstrained problems by deriving “constrained expressions”. Using these expressions, the governing physical models are modified and solved in the space without constraints. Below, we present a brief overview. For univariate functions, Ref. [1] introduced the constrained expression using the form,

(1) $$y(x)=g(x)+\sum_{k=1}^{n}\eta_{k}h_{k}(x),$$

where the *h*_*k*_(*x*) are *n* assigned linearly independent functions, *g*(*x*) is a free function, and the coefficients η_*k*_ are derived by imposing the *n* constraints. The resulting constrained expression always satisfies the constraints, as long as *g*(*x*) is defined, where the constraints are defined.

As an example, we consider a function *y*(*x*) subject to the constraints *y*(*x*_1_) = *y*_1_ and $y^{\prime }\left(x_{2}\right)=y_{2}^{\prime }$. Following Equation (1), and selecting *h*_1_(*x*) := 1 and *h*_2_(*x*) := *x*, the following two expressions are obtained [3,4].

$$y_{1}=g_{1}+\eta_{1}+\eta_{2}x_{1}\;y_{2}^{\prime }=g_{2}^{\prime }+\eta_{2}$$

where *g*_1_ and $g_{2}^{\prime }$ are values of the free function and its derivatives evaluated at the two constraint points *x*_1_ and *x*_2_. The unknowns *η*_1_ and *η*_2_ can be solved leading to,

$$\begin{array}{l} \eta_{1}=y_{1}-g_{1}-\left(y_{2}^{\prime }-g_{2}^{\prime }\right)x_{1}\\ \eta_{2}=y_{2}^{\prime }-g_{2}^{\prime }. \end{array}$$

Plugging the *η* coefficients back into Equation (1) and simplifying, the final constrained expression is produced,

$$y(x)=g(x)+\left(y_{1}-g_{1}\right)+\left(y_{2}^{\prime }-g_{2}^{\prime }\right)\left(x-x_{1}\right).$$

It can be seen that the constraints are always satisfied (*y*(*x*_1_) = *y*_1_ and $y^{\prime }\left(x_{2}\right)=y_{2}^{\prime }$), as long as *g*(*x*_1_) and $g^{\prime }\left(x_{2}\right)$ are defined. This is an example that demonstrates Equation (1). The theory is general and can be used for many other constraints. In the paper, we will present other examples (see also [1,3] for more examples).

In general, the *η*_*k*_ coefficients are expressed in terms of the independent variables and in terms of *g*(*x*) evaluated where the constraints are defined. The Equation (1) is called constrained expression because it represents *all* possible functions satisfying the *n* constraints, regardless of the function *g*(*x*). Please note that *g*(*x*) can be discontinuous, partially defined, and even the Dirac delta function, as long as there are no constraints specified where the delta function is infinite.

The function *y*(*x*) in Equation (1) typically is applied to ODEs or some variational problems. In general, in the example of ODEs, we can consider a problem for *y* = *y*(*x*),

$$F\left(x,y,y^{\prime },\ldots ,y^{\left(n\right)}\right)=0,$$

subject to the constraints, which can include various types of constraints, (e.g., constraints on the function or derivatives, integral constraints, or so on). Here, *F* is some equation of *y*(*x*) and its derivatives. Using a ToC expression (Equation (1)), this equation is transformed to an unconstrained problem where the unknown parameter is defined by a new function *g* = *g*(*x*). This transformation can be written as,

(2) $$\tilde{F}\left(x,g,g^{\prime },\ldots ,g^{\left(n\right)}\right)=0,$$

where $\tilde{F}$ is equations completely independent of the problem constraints. Moreover, $\tilde{F}$ differs from *F* and represents modified constitutive relations. The major advantage of Equation (2) is that it can be solved as an unconstrained optimization problem. Past work has focused on expanding the function of *g*(*x*) by a linear basis [5–7] such that

$$g(x)=\xi^{\text{T}}h(x),$$

where ***h***(*x*) is a vector of global functions and ***ξ*** is vector of unknown coefficients used in the optimization process. These coefficients are determined by minimizing the residual of Equation (2) at some collocation points [8,9]. In general, one can use locally supported ***h***(*x*) functions. One can also avoid a linear functional representation of *g*(*x*) and use a non-linear representation, for example, neural networks and support vector machines [10].

The Multivariate Theory of Connections [2] extends the original univariate theory [1] to *n*-dimensions and to any-degree boundary constraints. This extension can be summarized by the expression,

y(x)=A(c(x))+g(x)−A(g(x)),

where ***x*** = {*x*_1_, *x*_1_, …, *x*_*n*_} is the vector of *n* orthogonal coordinates, *c*(***x***) is a function specifying the boundary constraints, *A*(*c*(***x***)) is *any* interpolating function satisfying the boundary constraints, and *g*(***x***) is the free function. Several examples of constrained expressions can be found in Refs. [1,2].

The major research effort of Theory of Connections has been applied to solve linear [4] and non-linear [3] ODEs. This has been done by expanding the free function, *g*(*x*), in terms of a set of basis functions (e.g., orthogonal polynomials, Fourier, etc.). Linear or iterative non-linear least-squares is then used to solve for the coefficients of this expansion. This approach to solve ODEs/PDEs has many advantages over traditional methods: (1) it consists of a unified framework to solve IVP, BVP, or multi-values problems, (2) it provides an approximated solution expressed via analytical functions that can be used for subsequent algebraic manipulation, (3) the solution accuracy is usually obtained fast for many application problems, (4) the procedure can be numerically robust (very low condition number), and (5) it can solve ODE/PDE subject to a variety of constraint types: absolute, relative, linear, non-linear, and integral. Additionally, this technique has recently been extended to solve 2-dimensional PDEs [10].

The purpose of this paper is to introduce a set of five new applications of Theory of Connections; applications that are not covered in the previous references and where ToC is found effective. The main motivation and a short rational description of the new applications considered in this paper are:

1. **Analytic linear constraint optimization.** In this application, the analytical problem of finding the extreme of a quadratic vectorial form subject to linear constraint is obtained using ToC instead of using the classical Lagrange multipliers technique.
2. **Brachistochrone problem.** This application is dedicated to one of the most famous problem in calculus of variations: the brachistochrone problem [11]. This problem is solved numerically and at machine error accuracy via the two-point boundary-value problem of the Euler-Lagrange equation associated with the brachistochrone functional integral definition. In particular, the solution is obtained by deriving the differential equation in polar coordinates.
3. **Over-constrained differential equations.** This application takes into consideration the problem of solving ODEs subject to more constraints than the degree of the ODE which arise in many areas of science and engineering. For example, in the problem of orbit determination of a satellite the number of measurements exceeds the order of the governing differential equation [12]. Furthermore, multi-purpose optimization [13–15] deals specifically with optimizing across multiple objective functions simultaneous where trade-offs (weighting) between two conflicting objectives must be taken into account. In this section, a weight least-squares solution is provided for over-constrained differential equations by assigning relative weights to the constraints and then solving the weighted constrained ODE by ToC. Additionally, another example showing the sequence of continuous solutions of an IVP morphing into an BVP is provided.
4. **Inequality constraints.** This application extends the ToC framework to include inequality constraints [16]. This is obtained using a combination of sigmoid functions to keep the constrained expression within the inequality constraints. This allows for the derivation of functions constrained by user-defined bounds than can be asymmetric, continuous, or symmetric discontinuous functions. The main motivation for this constraint type comes from optimal control problems (bounded control inputs).
5. **Triangular domains.** Validated by mathematical proof, Ref. [2] has extended the original univariate theory [1] to the multivariate theory [2]. This extension represents the multivariate formulation of ToC subject to arbitrary-order derivative constraints in rectangular domains. This provides an analytical procedure to obtain constrained expressions in any orthogonal/rectangular space that can be used to transform constrained problems into unconstrained problems. In Ref. [2] particular emphasis and details are given to the 2-dimensional case, because of the most important applications to surfaces (PDEs, Topography, Visualization, etc.). This application begins the extension of ToC to triangular domains by deriving the surfaces satisfying boundary constraints.

The complete solution derivations of each one of these new applications, validated by numerical examples, are detailed in the subsequent sections.

## 2. Analytic Linear Constraints Optimization

This section shows an analytical application of the Theory of Connections. The problem, known as quadratic programming (QP) subject to equality constraints, consists of deriving a closed-form solution of the problem to find the min/max of the quadratic function, *F*(*x*), subject to *m < n* linear constraints,

(3) $$F(x)=\frac{1}{2}x^{\text{T}}Qx+c^{\text{T}}x\;\text{subject}\;\text{to}:\;Ax=b,$$

where $Q\in {\mathbb{R}}^{n\times n}$ and $Q=Q^{T}>0,c\in {\mathbb{R}}^{n},A\in {\mathbb{R}}^{m\times n}$, and $b\in {\mathbb{R}}^{m}$, are all assigned, and *D* has rank *m* (all rows are independent). Let us search a solution in the form,

$$x=g+A^{\text{T}}\eta ,$$

The constraint implies,

$$A\left(g+A^{\text{T}}\eta \right)=b\;\to \;AA^{\text{T}}\eta =b-Ag,$$

and

$$\eta ={\left(AA^{\text{T}}\right)}^{-1}b-{\left(AA^{\text{T}}\right)}^{-1}Ag.$$

Therefore,

$$x=g+A^{\text{T}}{\left(AA^{\text{T}}\right)}^{-1}b-A^{\text{T}}{\left(AA^{\text{T}}\right)}^{-1}Ag=x_{0}+\left[I_{n\times n}-A^{\text{T}}{\left(AA^{\text{T}}\right)}^{-1}A\right]g=x_{0}+Dg.$$

Substituting this expression of ***x*** in Equation (3) we obtain,

$$F(g)=\frac{1}{2}{\left(x_{0}+Dg\right)}^{\text{T}}Q\left(x_{0}+Dg\right)+c^{\text{T}}\left(x_{0}+Dg\right).$$

The problem of finding the extreme of Equation (3) is now an unconstrained optimization problem. Stationary condition implies

(4) df(g)dg=0→Ag+d=0

where

$$A=\frac{1}{2}D^{\text{T}}\left(Q+Q^{\text{T}}\right)D\;\text{and}\;d=\frac{1}{2}D^{\text{T}}\left(Q+Q^{\text{T}}\right)x_{0}+D^{\text{T}}c.$$

In this example, we show how to transform a constrained optimization problem to unconstrained optimization problem and find the unconstrained *g*(*x*), which is the solution of Equation (4). This shows a simple application of Theory of Connections. Unfortunately, the matrix *A* is singular. However, Equation (4) is a consistent linear system. Therefore, the solution can be found in the null space of *A*. This is computed by the Moore-Penrose inverse matrix (pseudo-inverse). Let $A=C\text{Λ}C^{T}$ be the spectral decomposition of *A*, where the eigenvector matrix, *C*, is an orthogonal matrix because matrix *A* is symmetric. Therefore, the solution of the problem given in Equation (3) is,

x=x0−DCΛ∗CTd,

where the diagonal matrix $\Lambda^{*}$ contains the inverse of the nonzero eigenvalues and zero for zero eigenvalues.

## 3. Brachistochrone Problem

The brachistochrone problem is one of many problems arising from the calculus of variations. This body of work focuses on finding the extrema of functionals (mappings from functions to real numbers). Associated functions that maximize or minimize the functionals can be found using the Euler-Lagrange equation. In general, this equation produces an ODE which when solved produces the extremal function. The main application of ToC to the calculus of variation is solving the specific ODEs which can be highly complex.

Consider finding the function, *f*_*s*_(*x*), minimizing the integral,

(5) $$I\left(f_{s}\right)=\min\int_{a}^{b}F\left(x,f(x),f^{\prime }(x)\right)\text{d}x$$

where *x* ∈ [*a*, *b*], *f*(*x*) is an unknown function satisfying *f*(*a*) = *f*_*a*_ and *f*(*b*) = *f*_*b*_, and $f(b)=f_{b},\text{and}F\left(x,f(x),f^{\prime }(x)\right)$ is the functional. An extremal, *f*_0_(*x*), of the integral given in Equation (5) is the solution of the Euler-Lagrange equation,

(6) $$\frac{\partial F}{\partial f}\left(x,f_{0}(x),f_{0}^{\prime }(x)\right)=\frac{\text{d}}{\text{d}x}\left[\frac{\partial F}{\partial f^{\prime }}\left(x,f_{0}(x),f_{0}^{\prime }(x)\right)\right].$$

**Figure 1.**
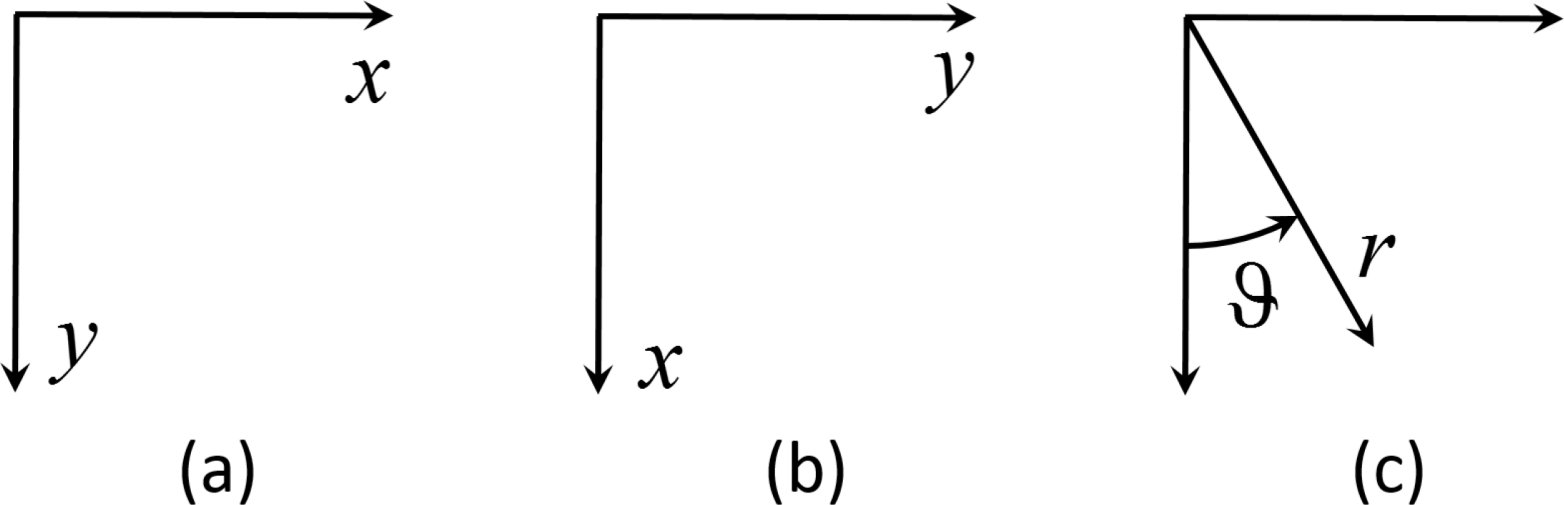
Brachistochrone Coordinate Systems: (**a**) classic cartesian coordinate system, (**b**) modified cartesian coordinate system, and (**c**) polar coordinate system.

The solution of this equation, *f*_0_(*x*), is an extremal solution (where the derivative is zero, which is a necessary condition). This solution, however, is not sufficient as *f*_0_(*x*) may be associated with some local minima, maxima, or saddle points. In other word, after solving Equation (6), we can consider *f*_*s*_(*x*) = *f*_0_(*x*), if the condition,

$$\int_{a}^{b}F\left(x,f_{0}(x),f_{0}^{\prime }(x)\right)\text{d}x\leq \min\int_{a}^{b}F\left(x,f(x),f^{\prime }(x)\right)\text{d}x,\;\forall \;f(x)$$

is verified. This can be done, for example, by evaluating the second derivative (Hessian) of $F\left(x,f(x),f^{\prime }(x)\right)$. Figure 1 shows three different coordinate systems that may be used to solve the brachistochrone problem.

Typically, the brachistochrone problem is written using coordinate system (a) of Figure 1. In this coordinate system, the total travel time of the sliding bead is given by the functional [11],

(7) $$F\left(x,y,y^{\prime }\right)=\sqrt{\frac{1+y^{\prime 2}}{2gy}},\;y=y(x)$$

subject to,

$$y(0)=0\;\text{and}\;y\left(x_{1}\right)=y_{1}.$$

Using the steps of the calculus of variations outlined in Equation (6) on the function in Equation (7) yields the differential equation for the function *y* = *y*(*x*),

(8) $$2yy^{\prime \prime }+y^{\prime 2}+1=0.$$

However, this coordinate system produces the case that $y^{\prime }(0)=\infty$ at the initial point and cannot be solved using ToC. If the axes are oriented as shown in coordinate system (b) of Figure 1, the functional used in the calculus of variations becomes,

$$F\left(x,y,y^{\prime }\right)=\sqrt{\frac{1+y^{\prime 2}}{2gx}},\;y=y(x)$$

which after performing the steps of the calculus of variations leads to the differential equation for the function *y* = *y*(*x*),

(9) $$2xy^{\prime \prime }+y^{\prime 3}+y^{\prime }=0.$$

The problem with the differential equation given in Equation (9) is that the solution may take on two different values of *y*(*x*) for a given value of *x*. Analytically, the issues with the DEs given in Equations (8) and (9) are circumvented by solving the problems parametrically. Unfortunately, the DE for the brachistochrone written in the parametric form either contains not-a-number or infinite values when implemented numerically, or admits a trivial solution. For example, let *x*(*p*) and *y*(*p*) each be functions of the parameter *p* and their derivatives with respect to *p* be given by the notation: $\text{d}x/\text{d}p=x_{p}\;\text{and}\;{\text{d}}^{2}x/\text{d}p^{2}=x_{pp}$. Then, Equation (9) can be rewritten using the chain rule as

(10) $$2x\frac{y_{pp}}{x_{pp}}+{\left(\frac{y_{p}}{x_{p}}\right)}^{3}+\frac{y_{p}}{x_{p}}=0,\;x=x(p),\;y=y(p).$$

Unfortunately, Equation (10) will have infinite values anytime *x*_*p*_ = 0 or *x*_*pp*_ = 0. Therefore, it cannot be implemented numerically. Alternatively, one could write the DE as

(11) $$2xy_{pp}x_{p}^{3}+x_{pp}\left(y_{p}x_{p}^{2}+y_{p}^{3}\right)=0,\;x=x(p),\;y=y(p).$$

However, Equation (11) admits the trivial solution *x* = 0. Attempts have been made to avoid the trivial solution by giving a starting value for the non-linear least-squares that is far from the solution *x* = 0, but none of these attempts were successful.

Another option is to express the brachistochrone problem in polar coordinates (coordinate system (c) in Figure 1). If the coordinates are chosen correctly, then the problems of the Cartesian coordinate systems can be avoided, and the problem does not need to be parameterized. A downside of this approach is a significantly more complicated DE; however, by using the ToC method, solving this DE is very easy. Using this coordinate system, the energy of an object sliding without friction is given by,

(12) $$\frac{1}{2}mv^{2}=mgr\;\sin\;\vartheta ,\;r=r(\vartheta ).$$

In addition, the differential path length in polar coordinates is given by,

(13) $$\text{d}s=\sqrt{r^{2}+r^{\prime 2}}\;\text{d}\vartheta ,$$

where $r^{\prime }=\text{d}r/\text{d}\vartheta$. Furthermore, using Equation (12), the velocity, *v*, can be written as,

(14) $$v=\frac{\text{d}s}{\text{d}t}=\sqrt{2gr\cos\vartheta }.$$

Using Equations (13) and (14) one can express the total time to get from the start point to the end point as

(15) $$T=\int_{0}^{t}\text{d}t=\int_{\vartheta_{0}}^{\vartheta_{f}}\frac{\sqrt{r^{2}+r^{\prime 2}}}{\sqrt{2gr\cos\vartheta }}\text{d}\vartheta .$$

Let the integrand of Equation (15) be defined as the functional $F\left(\vartheta ,r(\vartheta ),r^{\prime }(\vartheta )\right)$. Then, the calculus of variations can be used to minimize the travel time. This leads to the DE, after some simplification, given in Equation (16).

(16) $$-r^{2}(\dot{r}\tan\vartheta +2\ddot{r})-\tan\vartheta {\dot{r}}^{3}+3\dot{r}r+r^{3}=0,\;r=r(\vartheta ).$$

**Figure 2.**
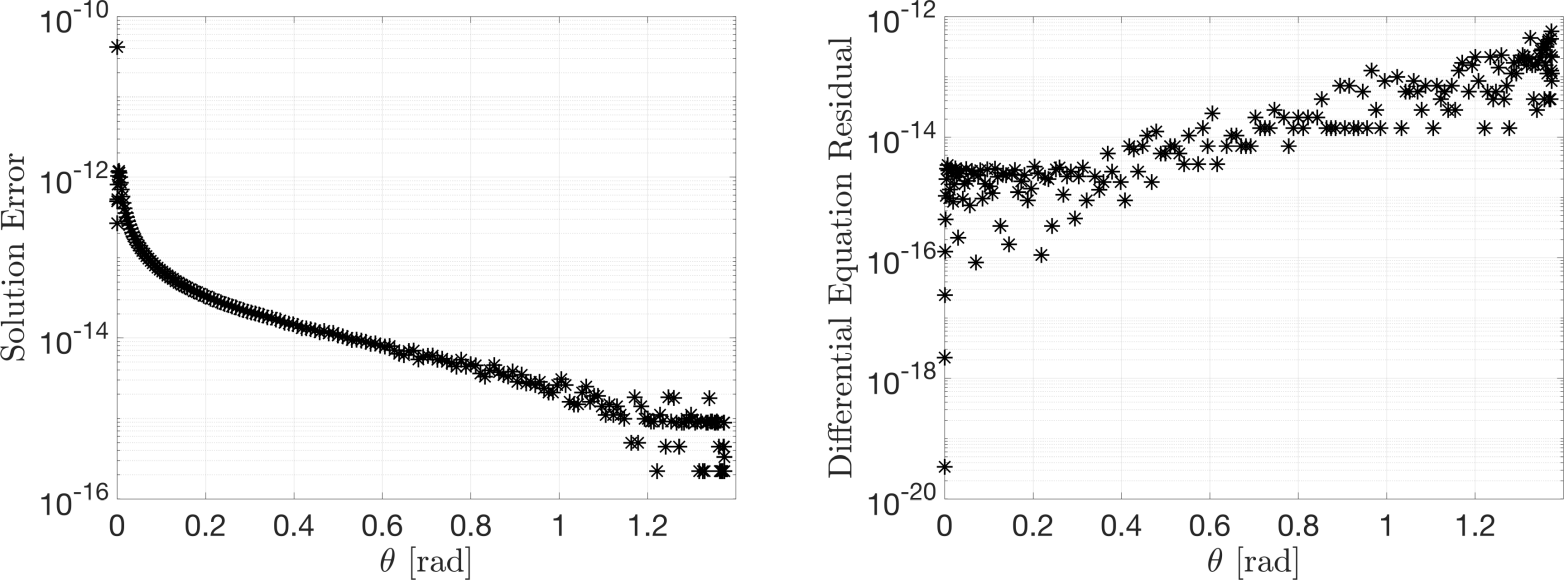
Polar brachistochrone differential equation solution error and residuals.

Using the differential equation given by Equation (16), the ToC method was used to solve the particular case with initial condition (*x*_0_, *y*_0_) = (0, 0) and final condition (*x*_*f*_, *y*_*f*_ ) = (1, 5). Figure 2 shows information associated with solving the brachistochrone problem and highlights the error between the ToC solution and the real solution. Additionally, the residuals of the DE are between the order of 10^−12^ and 10^−16^ when the ToC formulation is used and *g*(*x*) is expressed by 60 Chebyshev polynomials. It can be seen that this method can estimate the solution of the brachistochrone problem with high accuracy.

## 4. Over-Constrained Differential Equations

The seminal paper on ToC [1] presented Equation (1) as a way to incorporate *n* constraints to form a constrained expression, this equation is repeated below:

$$y(x)=g(x)+\sum_{k=1}^{n}\eta_{k}h_{k}(x).$$

This process leads to a (*n* × *n*) matrix populated by the values of *h*_*k*_(*x*) evaluated at the constraint conditions. The values of *η*_*k*_ can then be solve by simply inverting this matrix. Yet, through this process, it is *not* required that number of *η* coefficients equals the number of applied constraints, and thus constraint matrix can also be written for *n* values of η and *m* constraints. Which leads to,

$$M\eta =\left[\begin{array}{cccc} h_{1}^{\left(d_{1}\right)}\left(x_{1}\right) & h_{2}^{\left(d_{1}\right)}\left(x_{1}\right) & \ldots & h_{n}^{\left(d_{1}\right)}\left(x_{1}\right)\\ h_{1}^{\left(d_{2}\right)}\left(x_{2}\right) & h_{2}^{\left(d_{2}\right)}\left(x_{2}\right) & \ldots & h_{n}^{\left(d_{2}\right)}\left(x_{2}\right)\\ \vdots & \vdots & \ddots & \vdots \\ h_{1}^{\left(d_{m}\right)}\left(x_{m}\right) & h_{2}^{\left(d_{m}\right)}\left(x_{m}\right) & \ldots & h_{n}^{\left(d_{m}\right)}\left(x_{m}\right) \end{array}\right]\left\{\begin{array}{c} \eta_{1}\\ \eta_{2}\\ \vdots \\ \eta_{n} \end{array}\right\}=\left\{\begin{array}{c} y_{1}^{\left(d_{1}\right)}-g_{1}^{\left(d_{1}\right)}\\ y_{2}^{\left(d_{2}\right)}-g_{2}^{\left(d_{2}\right)}\\ \vdots \\ y_{m}^{\left(d_{m}\right)}-g_{m}^{\left(d_{m}\right)} \end{array}\right\},$$

where *d*_*i*_ denotes the order of derivative of the constraint at point *x*_*i*_ where *i* ∈ [1, *m*]. This becomes an over-determined system with the constraint matrix *M* being (*m* × *n*). The *η* values can still be solved by incorporating a weighted least-squares technique, where we define *W* as a diagonal weighting matrix,

$$W:=\left[\begin{array}{cccc} w_{1} & 0 & \ldots & 0\\ 0 & w_{2} & \ldots & 0\\ \vdots & \vdots & \ddots & \vdots \\ 0 & 0 & \ldots & w_{m} \end{array}\right],$$

leading to the solution,

$$\eta ={\left(M^{\text{T}}WM\right)}^{-1}M^{\text{T}}W\left\{\begin{array}{l} y_{1}^{\left(d_{1}\right)}-g_{1}^{\left(d_{1}\right)}\\ y_{2}^{\left(d_{2}\right)}-g_{2}^{\left(d_{2}\right)}\\ \vdots \\ y_{m}^{\left(d_{m}\right)}-g_{m}^{\left(d_{m}\right)} \end{array}\right\}.$$

**Figure 3.**
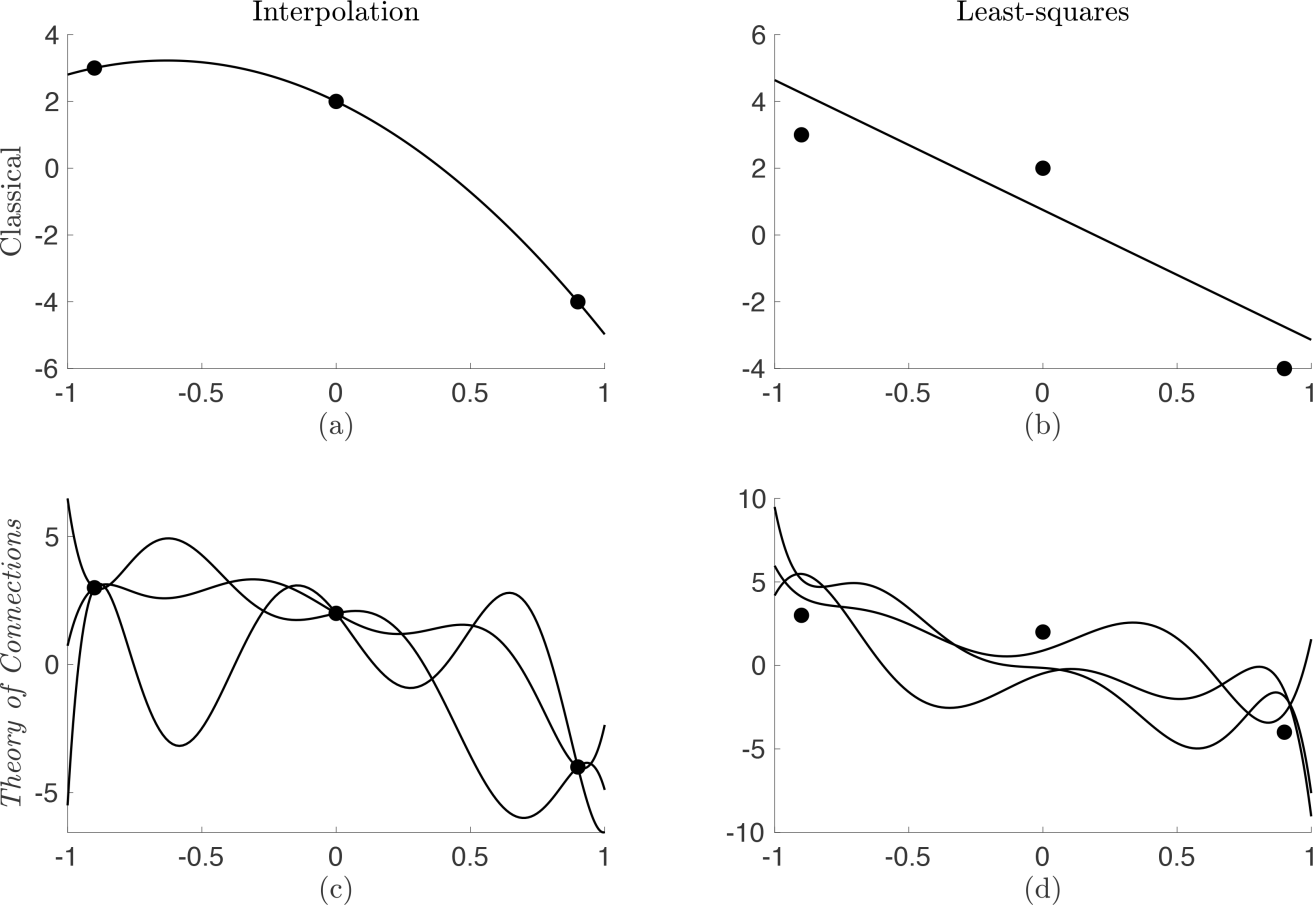
The classical formulation of (**a**) interpolation and (**b**) least-squares provides a single function solution. The ToC method provides the numerical framework to describe (**c**) all possible functions satisfying the constraints and (**d**) all possible functions of a least-squares fitting.

Unlike the traditional ToC approach where the *η* values produced an equation always satisfying the given constraints, the weight least-squares approach produces a weighted constrained expression which satisfies the constraints in a weighted (relative) sense. To exactly solve a Differential Equation, the number of constraints MUST be equal to the order of the Differential Equation. Yet, through the ToC method, the constrained expression is formed and *then* the differential equation is solved. This separation allows for the incorporation of more constraints than the order of the Differential Equation. The concept of this is highlighted in Figure 3 which provides an outline which distinguishes the ToC approach from classical methods in interpolation and least-squares. Part (c) in Figure 3 shows the original formulation of ToC where the number of the *η* coefficients was equal to the number of constraints incorporated. Part (d) highlights the work of this section which combines this general interpolation method with a weighted least-squares technique for the constraints.

### 4.1. Second Order Differential Equation with Three Point Constraints. A Numerical Example

As a numerical test, let us consider solving the Differential Equation,

(17) $$y^{\prime \prime }+2y^{\prime }+y=0,\;y=y(x).$$

For this example, let us assume that a dynamical system described by Equation (17) is “observed” at three points *x* = [−1, −0.5, +1] and these measurements are subject to normally distributed noise such that

$$y(-1)=N\left(y_{1}^{\text{true}},\sigma_{1}^{2}\right),\;y(-0.5)=N\left(y_{2}^{\text{true}},\sigma_{2}^{2}\right),\;\text{and}\;N\left(y_{3}^{\text{true}},\sigma_{3}^{2}\right),$$

where for this problem $y_{1}^{\text{true}}=5,y_{2}^{\text{true}}=4.515,\;\text{and}\;y_{3}^{\text{true}}=2$. For this, we define the weight matrix based on the variances,

$$W:=\left[\begin{array}{ccc} w_{1} & 0 & 0\\ 0 & w_{2} & 0\\ 0 & 0 & w_{3} \end{array}\right]=\left[\begin{array}{ccc} \sigma_{1}^{-2} & 0 & 0\\ 0 & \sigma_{2}^{-2} & 0\\ 0 & 0 & \sigma_{3}^{-2} \end{array}\right].$$

Using the development in the prior section, this Differential Equation can incorporate information from all three observations even though the Differential Equation is only second-order. Since the Differential Equation is second-order, the solution must be searched by the following constrained expression,

$$y(x)=g(x)+\eta_{1}h_{1}(x)+\eta_{2}h_{2}(x).$$

Then the values of *η*_1_ and *η*_2_ are computed by weighted least-squares where we define *h*_1_(*x*) := 1 and *h*_2_(*x*) := *x*,

$$\left\{\begin{array}{l} \eta_{1}\\ \eta_{2} \end{array}\right\}=\underset{V}{\underset{︸}{{\left(M^{\text{T}}WM\right)}^{-1}M^{\text{T}}W}}\left\{\begin{array}{l} y_{1}-g_{1}\\ y_{2}-g_{2}\\ y_{3}-g_{3} \end{array}\right\},\;M:=\left[\begin{array}{cc} 1 & x_{1}\\ 1 & x_{2}\\ 1 & x_{3} \end{array}\right]$$

where the *M* matrix is defined by imposing the constraints and the *W* matrix is populated by the scaled variances above. The solution leads to a constrained expression of the form,

(18) $$y(x)=g(x)+\beta_{1}(x)\left(y_{1}-g_{1}\right)+\beta_{2}(x)\left(y_{2}-g_{2}\right)+\beta_{3}(x)\left(y_{3}-g_{3}\right),$$

such that

$$\begin{array}{l} \beta_{1}(x)=w_{1}\left[w_{2}\left(x_{2}-x\right)\Delta_{21}+w_{3}\left(x_{3}-x\right)\Delta_{31}\right]/\left(\Delta_{21}^{2}w_{1}w_{2}+\Delta_{31}^{2}w_{1}w_{3}+\Delta_{32}^{2}w_{2}w_{3}\right)\\ \beta_{2}(x)=w_{2}\left[w_{1}\left(x-x_{1}\right)\Delta_{21}+w_{3}\left(x_{3}-x\right)\Delta_{32}\right]/\left(\Delta_{21}^{2}w_{1}w_{2}+\Delta_{31}^{2}w_{1}w_{3}+\Delta_{32}^{2}w_{2}w_{3}\right)\\ \beta_{3}(x)=w_{3}\left[w_{1}\left(x-x_{1}\right)\Delta_{31}+w_{2}\left(x-x_{2}\right)\Delta_{32}\right]/\left(\Delta_{21}^{2}w_{1}w_{2}+\Delta_{31}^{2}w_{1}w_{3}+\Delta_{32}^{2}w_{2}w_{3}\right). \end{array}$$

such that Δ_*ij*_ := *x*_*i*_ *− x*_*j*_. Equation (18) represents all functions satisfying constraints relative to the given variances $\sigma_{1}^{2},\sigma_{2}^{2},\text{and}\sigma_{3}^{2}$. With the constraints completely captured in Equation (18), the *g*(*x*) function represents the solution space that satisfies the three constraints by least-squares. Now let us move toward solving Equation (17). First, let the free function *g*(*x*), be expressed as a linear combination of a given basis set where *g*(*x*) = ***ξ***^T^***h***(*x*). Substituting this into Equation (18) leads to,

$$y(x)=\xi^{\text{T}}h(x)+\beta_{1}(x)\left(y_{1}-\xi^{\text{T}}h_{1}\right)+\beta_{2}(x)\left(y_{2}-\xi^{\text{T}}h_{2}\right)+\beta_{3}(x)\left(y_{3}-\xi^{\text{T}}h_{3}\right),$$

where the constrained expression can be reduced to the form,

$$y(x)=\xi^{\text{T}}a(x)+b(x),$$

**Figure 4.**
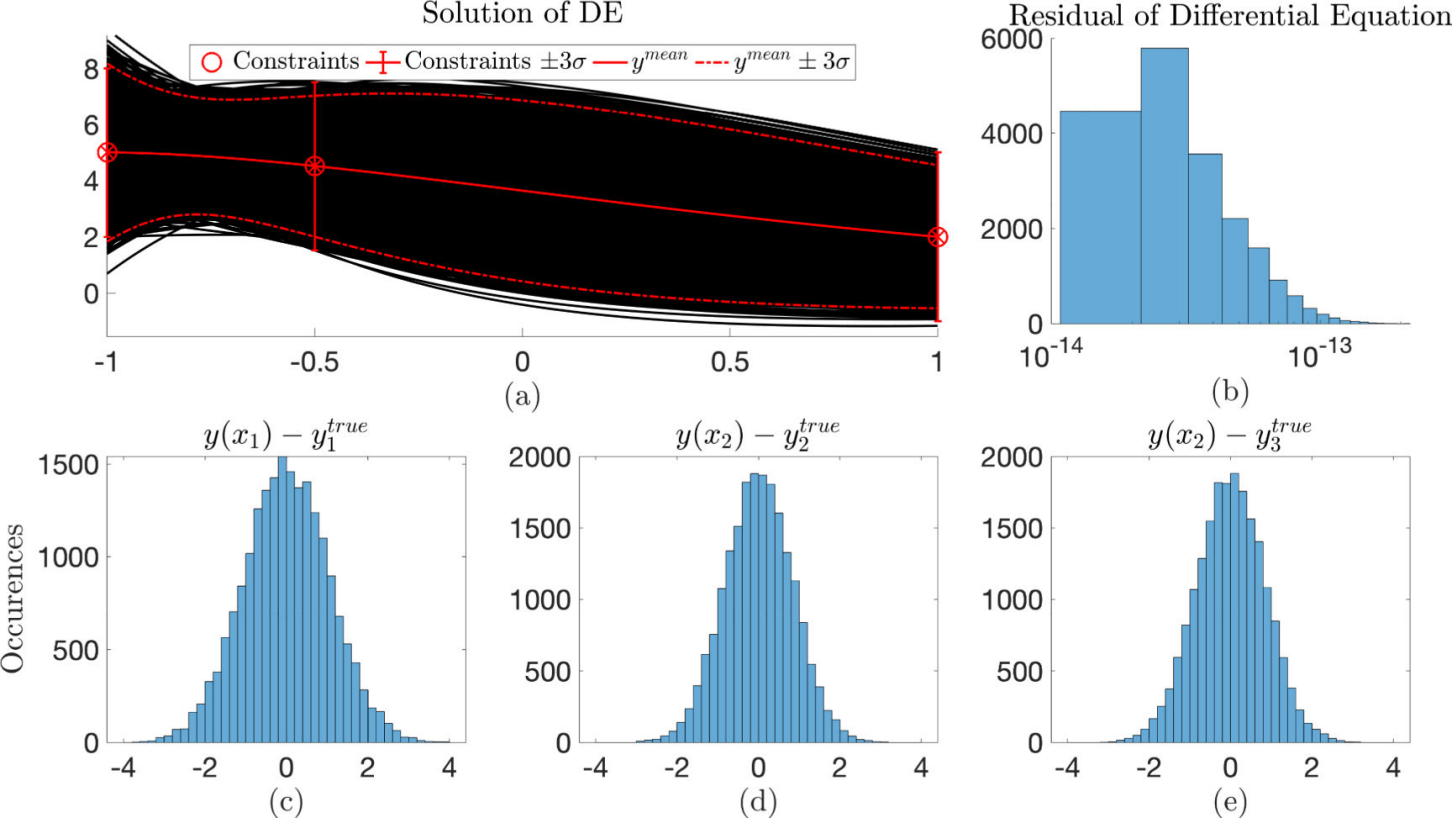
Monte Carlo test for 10,000 trials. Part (**a**) represents the solutions of the differential equation over the varying observed “constraints” with part (**b**) quantifying the accuracy of the solution. Parts (**c**)–(**e**) represent the distribution of the solution values compared with the true value of the “constraints”.

This form now has transformed the constrained optimization problem into an unconstrained optimization problem on ***ξ***, and can be solved using the techniques developed in Ref. [3,4]. In this specific problem, a Monte Carlo simulation of 10,000 trials was conducted to determine space that the function *y*(*x*) could occupy given the “observation” uncertainty and subject to dynamics governed by the Differential Equation. Figure 4 highlights this solution. In this figure, plot (a) shows the Differential Equation solution space given the observation uncertainty and plot (b) highlights the residuals of the Differential Equation over the entire simulation. It can be seen that the residuals of all solutions are between 10^−13^ and 10^−14^. Plots (c), (d), and (e) display the distribution of the constraint points around the true value. Note: these values are sampled from the solutions of the Differential Equation and not the constraints specified in the constrained expression.

Through this test, a probability bound for the Differential Equation can be produced, along with an estimated mean. For all solutions, the residual of the differential remained less than 10^−13^ verifying the accuracy of the method. Additionally, an interesting result of this test is in the final estimated solutions. Most evident in the constraints at *x* = −0.5 and *x* = +1, the 3*σ* of the Differential Equation is less than that of the observation 3*σ*. This arises because the loss function in the ToC method minimizes the residuals of the Differential Equation, and now in the over-constrained ToC method the residuals are minimized simultaneously with the weighted least-squares of the observations (or constraints).

### 4.2. Initial to Boundary-Value Problem Transformation. A Numerical Example

Another application made possible by this extension is a continuous transformation of an initial value problem to a boundary-value problem. The following numerically validates this approach. Consider solving,

$$y^{\prime \prime }+\left[\cos\left(3x^{2}\right)-3x+1\right]y^{\prime }+\left[6\sin\left(4x^{2}\right)-e^{\cos(3x)}\right]y=2\frac{\left[1-\sin(3x)](3x-\pi \right)}{4-x},\;y=y(x),$$

subject to the three constraints

$$y_{0}=y(-1)=-2,\;y_{0}^{\prime }=y^{\prime }(-1)=-2,\;\text{and}\;y_{f}=y(+1)=2.$$

Since the Differential Equation is second-order, the constrained expression takes the form,

$$y(x)=g(x)+\eta_{1}h_{1}(x)+\eta_{2}h_{2}(x).$$

Since the highest order constraint is the first derivative, we can safely define *h*_1_(*x*) := 1 and *h*_2_(*x*) := *x*. Then the values of *η*_1_ and *η*_2_ are computed again by weighted least-squares,

$$\left\{\begin{array}{l} \eta_{1}\\ \eta_{2} \end{array}\right\}=\underset{V}{\underset{︸}{{\left(M^{\text{T}}WM\right)}^{-1}M^{\text{T}}W}}\left\{\begin{array}{l} y_{0}-g_{0}\\ y_{0}^{\prime }-g_{0}^{\prime }\\ y_{f}-g_{f} \end{array}\right\},$$

where the *M* matrix is defined by imposing the constraints and the *W* matrix is a diagonal matrix of weights, i.e.,

$$M:=\left[\begin{array}{rr} 1 & -1\\ 0 & 1\\ 1 & 1 \end{array}\right]\;\text{and}\;W:=\left[\begin{array}{ccc} 1 & 0 & 0\\ 0 & 1-\alpha & 0\\ 0 & 0 & \alpha \end{array}\right],$$

and *α* is a weight parameter such that *α* ∈ [0, 1], which is synonymous with the transformation from the IVP to the BVP. From this derivation, *V* becomes

$$V=\frac{1}{1+4\alpha -\alpha^{2}}\left[\begin{array}{ccc} \alpha +1 & {\left(1-\alpha \right)}^{2} & 1+2\alpha -\alpha^{2}\\ -2\alpha & 1-\alpha^{2} & 2\alpha \end{array}\right].$$

and solving for *η* leads again Equation (18) of the form,

$$y(x)=g(x)+\beta_{1}(x)\left(y_{1}-g_{1}\right)+\beta_{2}(x)\left(y_{2}-g_{2}\right)+\beta_{3}(x)\left(y_{3}-g_{3}\right),$$

where the *β* terms are defined by,

$$\begin{array}{l} \beta_{1}(x)=(1+\alpha [1-2x]/\left(1+4\alpha -\alpha^{2}\right)\\ \beta_{2}(x)=\left({\left[1-\alpha \right]}^{2}+\left[1-\alpha^{2}\right]x\right)/\left(1+4\alpha -\alpha^{2}\right)\\ \beta_{3}(x)=\left(1+2\alpha [1+x]-\alpha^{2}\right)/\left(1+4\alpha -\alpha^{2}\right). \end{array}$$

**Figure 5.**
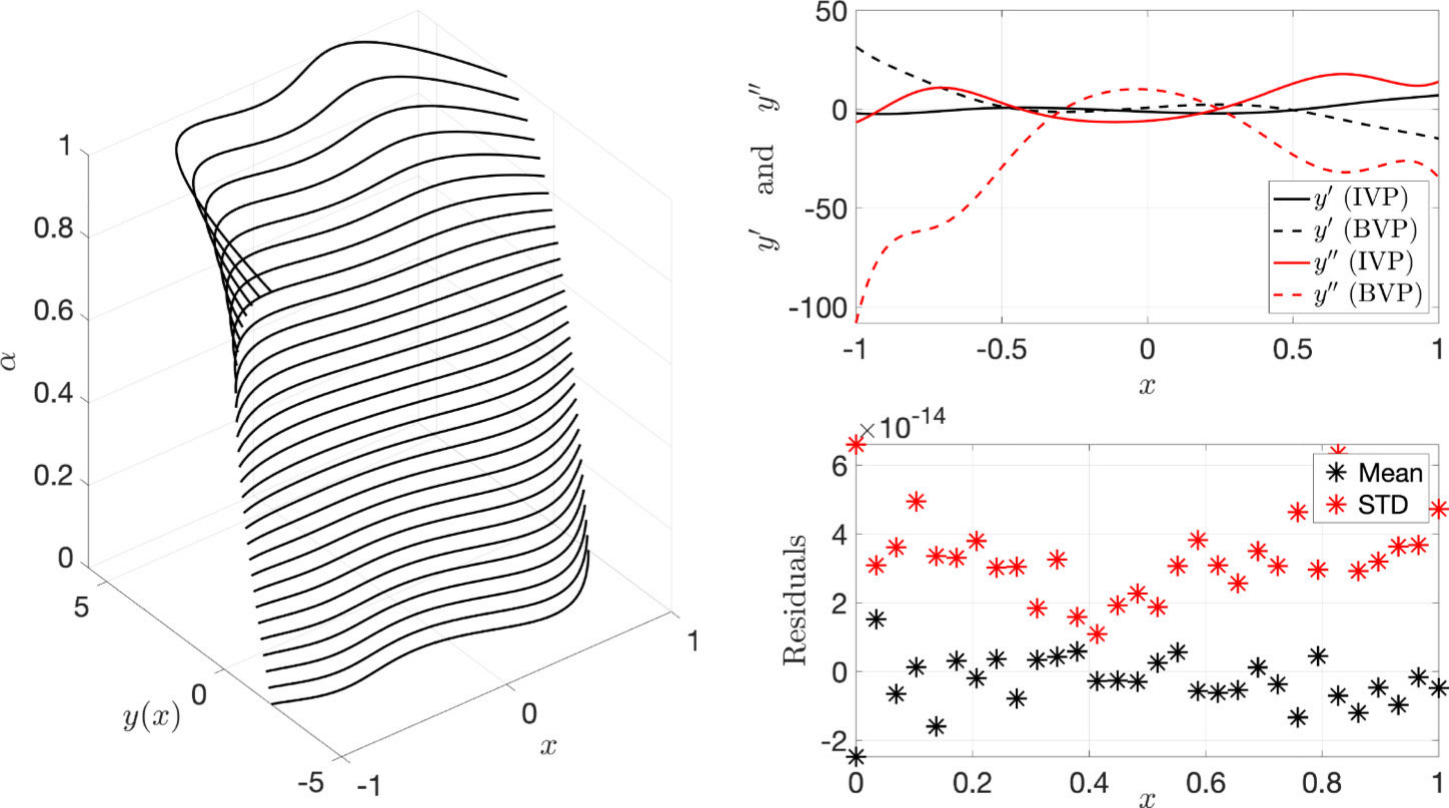
IVP to BVP Differential Equation parametric transformation.

Again, *g*(*x*) can be defined as *g* = _***ξ***_^T^***h***(*x*) and the unknown coefficient vector _***ξ***_ can be solved with least-squares. Figure 5 shows this transformation “surface” along with the residuals of the Differential Equation for validation of the method. It can be seen that the mean residual overall *α* values are on the order of 10^−14^ with a standard deviation on the same order. This plot shows the solution of the Differential Equation, *y*(*x*), continuously morphing from initial point and derivative constraints to initial and final point constraints.

## 5. Inequality Constraints

In some cases, it may be desired to find all possible trajectories between two functions, *f*_*u*_(*x*) and *f*_*d*_(*x*), that represent upper and lower continuous bounds, respectively. This means that *f*_*u*_(*x*) > *f*_*d*_(*x*), ∀ *x* ∈ [*x*_min_, *x*_max_]. All trajectories between these two functions can be approximated by the following expression,

(19) $$y(x)=g(x)+\left[f_{}(x)-g(x)\right]\sigma \left(k,g(x)-f_{u}(x)\right)+\left[f_{d}(x)-g(x)\right]\sigma \left(k,f_{d}(x)-g(x)\right)$$

where *g*(*x*) represents the free function. In the cases that there are also equality constraints to be applied, the function *g*(*x*) can be replaced by Equation (1). Additionally,

$$\sigma (k,z)=\frac{1}{1+e^{-kz}}\;\text{and}\;\frac{\text{d}\sigma (k,z)}{\text{d}z}=k\sigma (k,z)(1-\sigma (k,z)),$$

is a sigmoid function with smoothing parameter *k*. The sigmoid function acts as a “switching function” that subtracts off the portion of *g*(*x*) that exceeds the bounds. In this equation, *k* is not infinite, and thus *y*(*x*) will experience some overshooting/undershooting when *g*(*x*) > *f*_*u*_(*x*) or *g*(*x*) < *f*_*d*_(*x*) that must be quantified. To analyze this, let us consider a simple case where the function is only constrained from above with *f*_*u*_(*x*) and therefore only overshooting exists.

(20) $$y(x)=g(x)+\left[f_{u}(x)-g(x)\right]\sigma \left(k,g(x)-f_{u}(x)\right).$$

**Figure 6.**
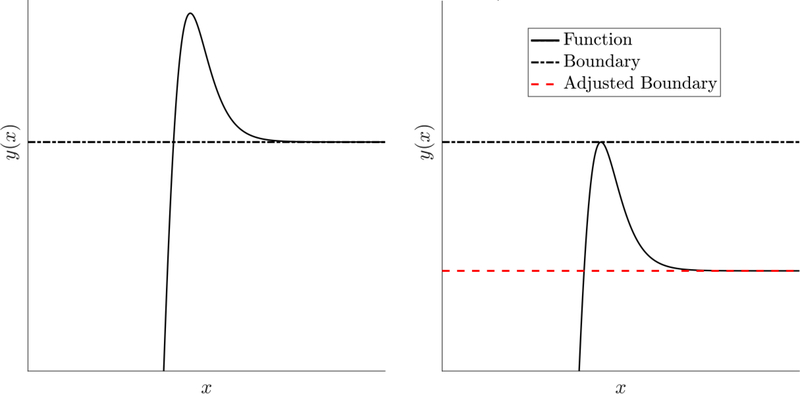
Overshoot of *y*(*x*) for the upper bound constraint. This phenomenon can be quantified and can be used to eliminate the overshooting.

For this case, the overshooting phenomenon is shown in Figure 6.

Since the only free part of the equation is *g*(*x*), the maximum value of the entire function, *y*(*x*), will be when the derivative ( d*y*(*x*)/ d*g*(*x*) = 0). Performing this derivative, Equation (20) becomes,

$$\frac{\text{d}y}{\text{d}g}=0=1-\frac{1}{1+e^{-k\left(g(x)-f_{u}(x)\right)}}\frac{e^{-k\left(g(x)-f_{u}(x)\right)}k\left(f_{u}(x)-g(x)\right)}{(1+e^{{-k\left(g(x)-f_{u}(x)\right))}^{2}}},$$

which can be solved for *g*(*x*). The resulting value, *g*(*x*)_max_, is the value of *g*(*x*) that will cause the maximum overshoot in *y*(*x*). This process yields,

$$g{\left(x\right)}_{\max}=\frac{kf_{u}(x)+\mathrm{productlog}\left(e^{-1}\right)+1}{k}$$

where productlog is the product logarithm function (also-called the omega function or the Lambert *W* function). Substituting this result back into Equation (19), we can obtain the maximum value that *y*(*x*) can take.

(21) $$y{\left(x\right)}_{\max}=f_{u}(x)+\frac{\mathrm{productlog}\left(e^{-1}\right)}{k}.$$

Now, we use Equation (21) to solve for a modified upper bound, ${\hat{f}}_{u}(x)$, such that the maximum value of *y*(*x*) is $f_{u}(x):{\hat{f}}_{u}(x)$ is the adjusted boundary shown in Figure 6. This yields Equation (22).

(22) $${\hat{f}}_{u}(x)=f_{u}(x)-\frac{\text{productlog}\;\left(e^{-1}\right)}{k}.$$

A similar derivation can be made for the lower bound ${\hat{f}}_{d}(x)$ ; the solution is shown in Equation (23).

(23) $${\hat{f}}_{d}(x)=f_{d}(x)+\frac{\text{productlog}\left(e^{-1}\right)}{k}.$$

The functions ${\hat{f}}_{u}(x)$ and ${\hat{f}}_{d}(x)$ replace *f*_*u*_(*x*) and *f*_*d*_(*x*) in Equation (19), resulting in,

(24) $$y(x)=g(x)+\left[f_{u}(x)-g(x)\right]\sigma \left(k,g(x)-f_{u}(x)\right)+\left[f_{d}(x)-g(x)\right]\sigma \left(k,f_{d}(x)-g(x)\right).$$

**Figure 7.**
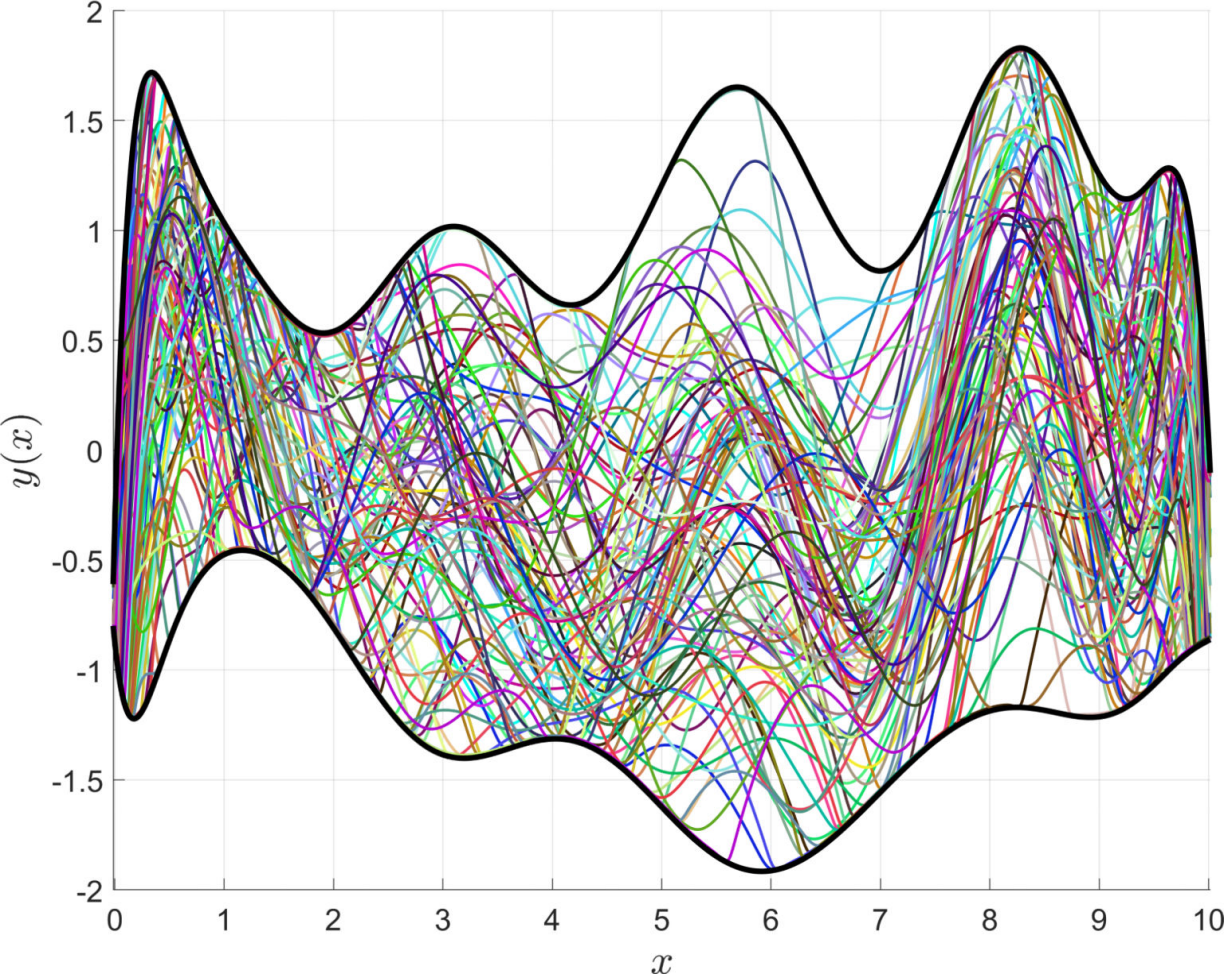
Random constrained expressions with continuous bounds.

Equation (21) shows that the value of *k* is the only user-selected parameter that effects the difference between *y*(*x*)_max_ and *f*_*u*_(*x*), because productlog(*e*^−1^) is a constant. Increasing the value of *k* will decrease the difference between *y*(*x*)_max_ and *f*_*u*_(*x*), and also the difference between *y*(*x*)_min_ and *f*_*d*_(*x*). Decreasing the value of *k* will have the opposite effect. Equation (24) was used to generate Figure 7, which contains randomly generated inequality boundaries and functions. This figure shows that all the random functions stay within the randomly generated inequality boundaries, and thus demonstrates how this technique can be used to enforce inequality boundary constraints.

## 6. Triangular Domains

**Figure 8.**
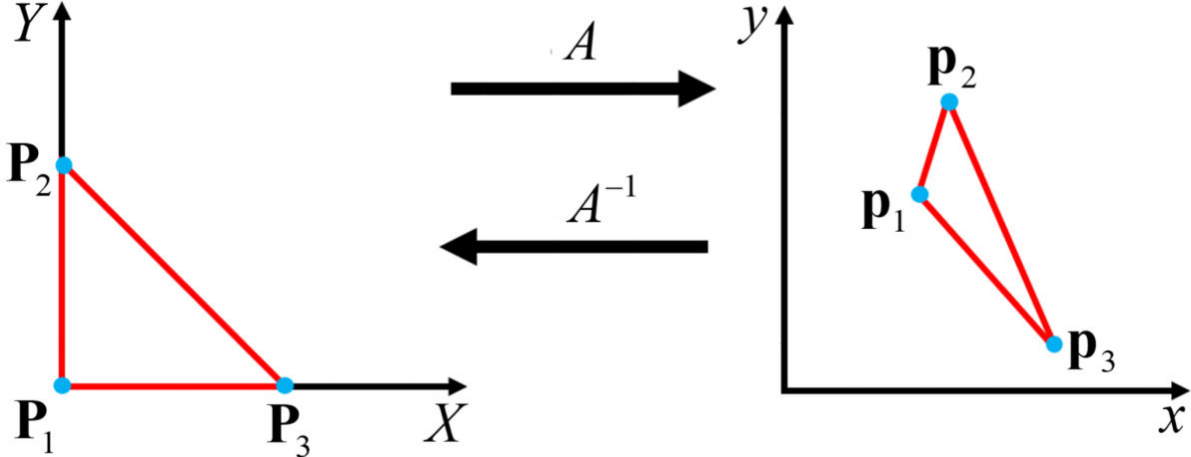
Affine Transformation from Unit Triangle to Desired Triangle.

This section demonstrates how to construct a constrained expression with a free function, *g*(*x*, *y*), that will always meet the Dirichlet boundary conditions on a triangular domain. This section is broken down into two subsections. The first proves that an affine transformation exists that can transform the unit triangle into any desired triangle: shown in Figure 8. The second subsection shows how to build the constrained expression for the unit triangle, and how to extend it, via the affine transformation given in the first subsection, to any triangular domain.

### 6.1. Affine Transformation from the Unit Triangle to the Generic Triangle

Consider a unit triangle, which is shown on the left plot of Figure 8. This unit triangle has the vertices defined by the vectors, ***P***_1_ := {0, 0}^T^, ***P***_2_ := {0, 1}^T^, and ***P***_3_ := {1, 0}^T^.

This subsection presents an affine transformation, denoted by A, that transforms the coordinates of a unit triangle defined in the [*X*,*Y*] (capital frame) into the coordinates [*x*, *y*, *z*] of the triangle in the desired coordinate frame.

Let the three vectors ***P***_*i*_, where *i* ∈{1, 2, 3}, be the position vectors of vertices of the unit triangle in the starred frame. Similarly, ***p***_*i*_, where *i* ∈ {1, 2, 3}, be the position vectors of the vertices of the desired triangle in the regular frame. Then, the affine coordinate transformation given in Equation (25) transforms the unit triangle in the capital frame into the desired triangle in the desired frame.

(25) $$\left\{\begin{array}{l} x\\ y\\ z \end{array}\right\}=\left[p_{3}-p_{1},\;p_{2}-p_{1},\;\left(p_{3}-p_{1}\right)\times \left(p_{2}-p_{1}\right)\right]\left\{\begin{array}{l} X\\ Y\\ 0 \end{array}\right\}+p_{1}=A\left\{\begin{array}{l} X\\ Y\\ 0 \end{array}\right\}+p_{1}.$$

The following identities prove the mapping provided by Equation (25),

$$p_{1}=A\left\{\begin{array}{l} 0\\ 0\\ 0 \end{array}\right\}+p_{1},\;p_{2}=A\left\{\begin{array}{l} 0\\ 1\\ 0 \end{array}\right\}+p_{1},\;\text{and}\;p_{3}=A\left\{\begin{array}{l} 1\\ 0\\ 0 \end{array}\right\}+p_{1}.$$

The inverse transformation is also of interest, and can be written as,

(26) $$\left\{\begin{array}{l} X\\ Y \end{array}\right\}=[\begin{array}{ll} p_{3}-p_{1}, & {p_{2}-p_{1},\;\left(p_{3}-p_{1}\right)\times \left(p_{2}-p_{1}\right)]}^{-1}\left(\left\{\begin{array}{l} x\\ y\\ z \end{array}\right\}-p_{1}\right) \end{array}.$$

The inverse transformation will always exist if the triangle is non-degenerate. The first column of *A* is the vector that points from *p*_3_ to *p*_1_, and the second column is the vector from that points from *p*_2_ to *p*_1_. For a triangle, these two vectors will never be parallel. The third column of *A* is defined as the cross-product of the first two columns, and will therefore be orthogonal to the vectors that make up the first two columns. Therefore, the columns of *A* are linearly independent, and *A* is invertible. Therefore, the inverse transform always exists.

### 6.2. Coons-Type Surface on the Unit Triangle

Consider unit triangle with boundary constraints provided by the boundary functions, *F*_1_(0,*Y*), *F*_2_(*X*, 1 − *X*) = *F*_2_(1 − *Y*,*Y*) when *Y* + *X* = 1, and *F*_3_(*X*, 0). For this unit triangle, the following Coons-type surface can be constructed,

(27) $$\begin{array}{l} F(X,Y)=(1-X-Y)\left[F_{3}(X,0)+F_{1}(0,Y)-F_{1}(0,0)\right]+\\ \;+X\left[F_{3}(X,0)-F_{3}(1-Y,0)+F_{2}(1-Y,Y)\right]+\\ \;+Y\left[F_{1}(0,Y)-F_{1}(0,1-X)+F_{2}(X,1-X)\right]. \end{array}$$

From Equation (27) one can show that

$$F(X,0)=F_{3}(X,0),\;F(0,Y)=F_{1}(0,Y),\;\text{and}\;\{\begin{array}{l} F(X,1-X)=F_{2}(X,1-X)\\ F(1-Y,Y)=F_{2}(1-Y,Y). \end{array}$$

Let’s write Equation (27) in the following compact form,

(28) $$F(X,Y)=C\left(F_{1},F_{2},F_{3}\right)$$

where *C*(*F*_1_, *F*_2_, *F*_3_) is a function of the boundary constraints, *F*_1_(0,*Y*), *F*_2_(*X*, 1 − *X*) = *F*_2_(1 − *Y*,*Y*), and *F*_3_(*X*, 0).

### 6.3. ToC Surfaces on the Unit Triangle

Equation (28) can be specified for any surface, *Z* = *G*(*X*,*Y*). This means that we can write the identity,

$$G(X,Y)=C\left(G_{1},G_{2},G_{3}\right)$$

where *G*_1_ = *G*(0,*Y*), *G*_2_ = *G*(*X*, 1 − *X*) or *G*_2_ = *G*(1 − *Y*,*Y*), and *G*_3_ = *G*(*X*, 0). Therefore, ToC allows us to write the constrained expression representing *all* surfaces satisfying the boundary conditions on the unit triangle as,

(29) $$F(X,Y)=C\left(F_{1},F_{2},F_{3}\right)+G(X,Y)-C\left(G_{1},G_{2},G_{3}\right)$$

where *G*(*X*,*Y*) is a free function. The constrained expression given in Equation (29) can be written explicitly as,

$$\begin{array}{cc} F(X,Y)= & G(X,Y)+(1-X-Y)\left[F_{3}(X,0)-G(X,0)+F_{1}(0,Y)-G(0,Y)-F_{1}(0,0)+G(0,0)\right]+\\ & X\left[F_{3}(X,0)-G(X,0)-F_{3}(1-Y,0)+G(1-Y,0)+F_{2}(1-Y,Y)-G(1-Y,Y)\right]+\\ & Y\left[F_{1}(0,Y)-G(0,Y)-F_{1}(0,1-X)+G(0,1-X)+F_{2}(X,1-X)-G(X,1-X)\right]. \end{array}$$

### 6.4. ToC Surfaces on the Generic Triangle

The ToC surfaces on the generic triangle can be derived using Equation (29) and the affine mapping given in Equation (26). Setting, ***X*** = (*X*,*Y*), Equation (29) can be rewritten as

$$F(X)=C\left[F_{1}(X),F_{2}(X),F_{3}(X)\right]+G(X)-C\left[G_{1}(X),G_{2}(X),G_{3}(X)\right].$$

Setting, ***x*** = (*x*, *y*, *z*), the affine transformation can be written as

$$X=A^{-1}\left(x-p_{1}\right),$$

and the ToC Surfaces on the Generic Triangle can be written as,

$$F(x)=C[F_{1}(X(x)),F_{2}((X(x))),F_{3}((X(x))]+G((X(x)))-C\left[G_{1}((X(x))),G_{2}((X(x))),G_{3}((X(x)))\right].$$

$$\begin{array}{l} F_{1}=(6-y)(y-1.5)\cos(3x)+4,\\ F_{2}=(2-x)(x-3)\exp(x)\cos(2y)+4,\\ F_{3}=(y-1.5)(x-3)\sqrt{xy}+4, \end{array}$$

and the vertices of the triangle were *P*_1_ = (1, 1.5), *P*_2_ = (2, 6), and *P*_3_ = (3, 1).

**Figure 9.**
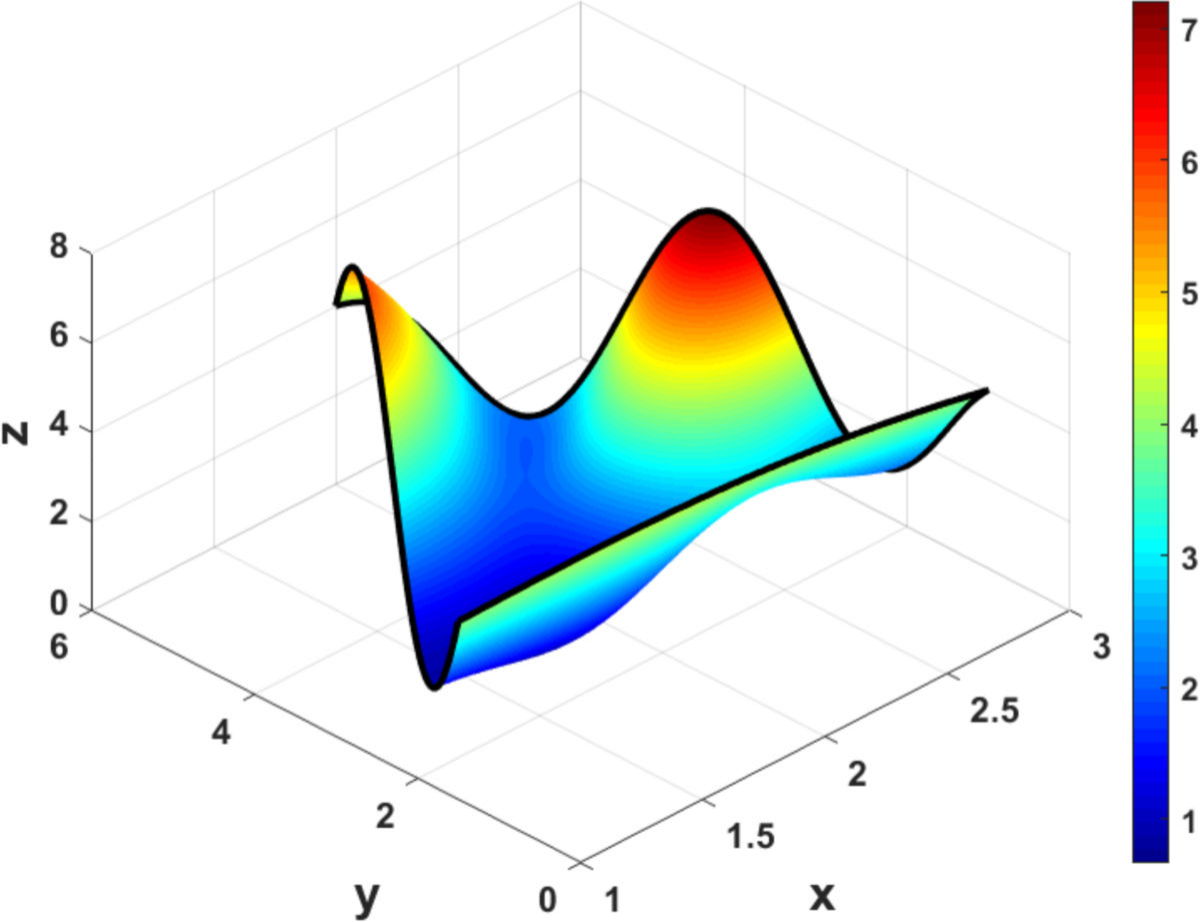
ToC Triangular Surface with *g*(*x*, *y*) = 0.

**Figure 10.**
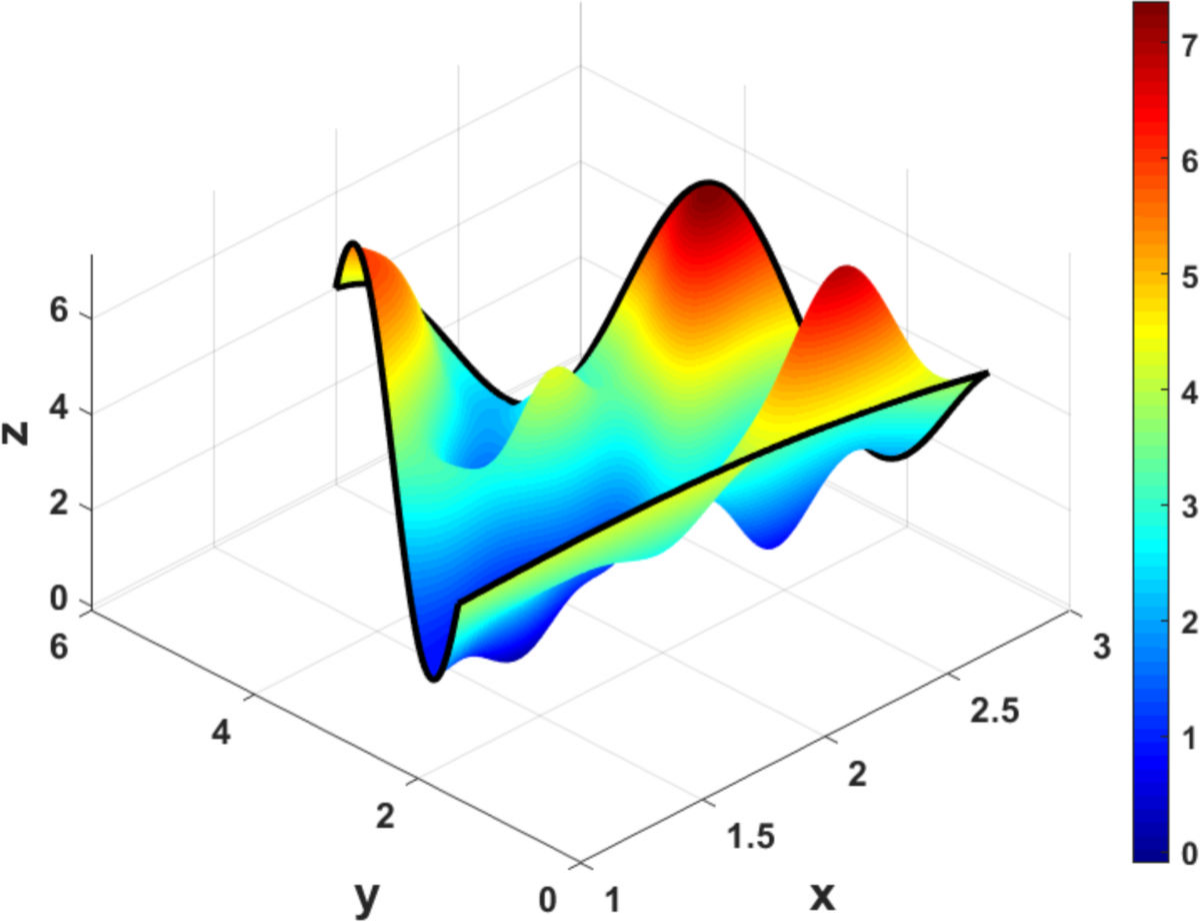
ToC Triangular Surface with $g(x,y)=\cos\left(\frac{1}{2}\exp x\right)\sin(5y)\sqrt{x}$.

Figures 9 and 10 show that the ToC surfaces meet the triangular boundary constraints (shown in black) regardless of the free function, *g*(*x*, *y*), chosen.

## 7. Conclusions

This work demonstrated some of the applications of ToC as a unified framework. The first application showed how to solve QP problems subject to equality constraints without the use of Lagrange multipliers. Using ToC, the equality constraints are embedded into a constrained expression that transforms the problem into an unconstrained optimization problem. The second application showed how to solve one of the most famous calculus of variations problems, the brachistochrone problem, with ToC. The calculus of variations technique was used to derive a highly non-linear differential equation in polar coordinates, to avoid singularities, which was solved via ToC. The third application explored solving over-constrained differential equations, where there are more constraints than the order of the differential equation. Using ToC through weighted least-squares, we transform the problem into unconstrained problem, which can easily be solved. The fourth application showed how to extend ToC to incorporate inequality constraints by using sigmoid functions. In addition, a discussion on avoiding undershoot/overshoot was included. The fifth and final application showed how to write constrained expressions for triangular domains subject to Dirichlet boundary conditions.

Future work will investigate how some of these applications can be extended to solve optimal control problems. The QP problem subject to linear equality constraints and solving the brachistochrone problem represent the first research thrust in that direction, as these are some of the classic problems in the field of optimal control. Moreover, future work will investigate how these applications can be combined with the application on inequality constraints to solve optimal control problems subject to inequality constraints.

Furthermore, future work will investigate applications of over-constrained differential equations. One example is applications in orbit determination where measurements exceed the order of the differential equation governing the dynamics of the orbiting body. By using the approach developed for weighted least-squares solutions of differential equations, the dynamics of the body and variance of observational measurements can be combined into a single optimization problem. Another area of future work includes multi-objective optimization problems.

Additionally, future research will look to extend constrained expressions on triangular domains to include constraints on the derivatives normal to the sides of the triangle. The obvious application of this research extension will be in solving differential equations on triangular domains; however, this research extension also has applications in meshing, domain discretization, and visualization.

## Abbreviations

The following abbreviations are used in this manuscript:

BVP: boundary-value problem
DE: differential equation
IVP: initial value problem
ODE: ordinary differential equation
PDE: partial differential equation
QP: quadratic programming
ToC: Theory of Connections

## References

[1] Mortari D The Theory of Connections: Connecting Points. Mathematics 2017, 5, 57.

[2] Mortari D; Leake C The Multivariate Theory of Connections. Mathematics 2019, 7, 296. 10.3390/math7030296 PMC7259476 32477923

[3] Mortari D; Johnston H; Smith L High accuracy least-squares solutions of nonlinear differential equations. J. Comput. Appl. Math. 2019, 352, 293–307. 32454554 10.1016/j.cam.2018.12.007 PMC7243685

[4] Mortari D Least-squares Solution of Linear Differential Equations. Mathematics 2017, 5, 48.

[5] Gottlieb D; Orszag SA Numerical Analysis of Spectral Methods: Theory and Applications; Society for Industrial and Applied Mathematics: Philadelphia, PA, USA, 1977.

[6] Jain MK Numerical Solution of Differential Equations; Wiley Eastern: New Delhi, India, 1979.

[7] Driscoll TA; Hale N Rectangular spectral collocation. IMA J. Numer. Anal. 2016, 36, 108–132.

[8] Lanczos C Applied Analysis In Progress in Industrial Mathematics at ECMI 2008; Dover Publications, Inc: New York, NY, USA, 1957; Chapter 7, p. 504.

[9] Wright K Chebyshev Collocation Methods for Ordinary Differential Equations. Comput. J 1964, 6, 358–365.

[10] Leake C; Johnston H; Smith L; Mortari D Approximating Ordinary and Partial Differential Equations Using the Theory of Connections and Least Squares Support Vector Machines. Trans. Neural Netw. Learn. Syst 2019, submitted.

[11] Hurtado JE Paul Bunyan’s Brachistochrone and Tautochrone. J. Astronaut. Sci. 2000, 48, 207–224.

[12] Bate RR; Mueller DD; White JE Fundamentals of Astrodynamics; Dover Publications, Inc.: New York, NY, USA, 1971; Chapter 2.

[13] Miettinen K Nonlinear Multiobjective Optimization In International Series in Operations Research & Management Science; Springer: New York, NY, USA, 1999.

[14] Shirazi A; Najafi B; Aminyavari M; Rinaldi F; Taylor RA Thermal economic environmental analysis and multi-objective optimization of an ice thermal energy storage system for gas turbine cycle inlet air cooling. Energy 2014, 69, 212–226.

[15] Ellefsen K; Lepikson H; Albiez J Multiobjective coverage path planning: Enabling automated inspection of complex, real-world structures. Appl. Soft Comput. 2017, 61, 264–282.

[16] Lewis FL; Vrabie DL; Syrmos VL Optimal Control; John Wiley & Sons, Inc.: Hoboken, NJ, USA, 2012.

